# Immigrants’ diminished protective effects of parental education and employment on youth mood disorders in Sweden

**DOI:** 10.1186/s12888-025-07264-7

**Published:** 2025-08-20

**Authors:** Shervin Assari, Mehdi Osooli, Henrik Ohlsson, Jan Sundquist, Kristina Sundquist

**Affiliations:** 1 Department of Internal Medicine, Charles R Drew University of Medicine and Science, Los Angeles, CA USA; 2 Department of Family Medicine, Charles R Drew University of Medicine and Science, Los Angeles, CA USA; 3 Department of Urban Public Health, Charles R Drew University of Medicine and Science, Los Angeles, CA USA; 4 Marginalization Related Diminished returns, Los Angeles, CA USA; 5 Division Clinical Epidemiology and Center for Pharmacoepidemiology, Department of Medicine Solna, Karolinska Institutet, Stockholm, Sweden; 6 Center for Primary Health Care Research, Department of Clinical Sciences, Lund University, Malmö, Sweden; 7 University Clinic Primary Care, Skåne University Hospital, Region Skåne, Sweden

**Keywords:** Immigrant, Mood disorders, Inequity, Young adult, Socioeconomic status, Sweden, First-generation, Second-generation

## Abstract

**Objectives:**

While high socioeconomic status (SES) indicators generally protect against mental health issues such as mood disorders in overall populations, evidence suggests that these protective effects might be attenuated for marginalized groups such as immigrants, in comparison to groups who are more socially privileged. This phenomenon is referred to as Marginalization-related Diminished Returns (MDRs). Existing knowledge on diminished returns of SES indicators primarily stems from cross-sectional comparisons of racial groups of adults in the US. Therefore, there is a crucial need to investigate these dynamics using longitudinal data of youth in European countries that are host countries for migrants from many different countries.

**Aims:**

This study aims to compare the impact of family SES indicators (parental education, parental employment, family income), and family composition on subsequent incidence of mood disorders in youth from immigrant and non-immigrant families.

**Methods:**

Utilizing a retrospective cohort design, we analyzed data from nationwide registers in Sweden from Jan 1, 2001, to Dec 31, 2020, and included immigrant and non-immigrant children 12–18 years. Independent variables included parental education, parental employment, family income, and family composition. The outcome variable was a mood disorder diagnosis based on the national patient register. Sex and study year served as covariates, while immigration background acted as a moderator. Cox regression models were employed for statistical analysis.

**Results:**

All SES indicators (higher parental education, parental employment, higher family income), and two-parent family composition were associated with a lower risk of mood disorders. However, the protective effects of parental education and employment were weaker for immigrant youth compared to native-born youth, as documented by statistically significant interaction tests.

**Conclusions:**

Our findings reveal weaker protective effects of parental education and employment for youth from immigrant families compared to youth from native-born families in Sweden. Prevention of mood disorders among immigrant families in Sweden may require two sets of policy interventions: one that supports education and employment in general and one that supports employed and educated families to harness their available human capital, with particular attention to immigrants from Africa and the Middle East.

**Supplementary Information:**

The online version contains supplementary material available at 10.1186/s12888-025-07264-7.

## Introduction

High family socioeconomic status (SES) indicators protect family members including youth against a broad spectrum of health problems [1, 2]. Youth from families with higher education, employment, higher income, and two parents tend to report lower levels of mood disorders, such as anxiety and depression [3]. In contrast, youth from families that have less education, unemployment, lower income, and one parent are generally at an increased risk of mood disorders [4].

However, according to the Marginalization-related Diminished Returns (MDRs) theory [5]the health effects of family and individual SES indicators are less pronounced for socially marginalized populations [6]. A growing body of research suggests that belonging to any socially marginalized group may be associated with attenuated protective effects of SES indicators on youth health outcomes [7]. Most of this research, however, originates from the United States [8]and very few studies have replicated these patterns in Europe [9]. Moreover, the existing studies predominantly focus on the effects of race/ethnicity rather than immigration as source of social marginalization [10]. Finally, a large body of these studies are cross-sectional [5]. Thus, there is a need to investigate whether the protective influence of family SES indicators on mood disorders in youth differs between immigrant and native-born families in follow-up studies in European settings, where the diversity has increased over time due to global migration.

As mentioned earlier, if social marginalization weakens the positive association between family SES indicators and health, then youth from high SES families with marginalized identities will still experience higher-than-expected (given their SES) rates of mental health problems [11]. In the US, the SES gradient in health (i.e., positive association between SES and health) is shown to be weaker for Black [11]Hispanic [12]Asian American [13]and Native American [14]and immigrant [9] individuals, compared to non-Latino white, native-born US citizens.

Considering the geographic proximity and perceived safety of Europe, many refugees have chosen to immigrate to European countries, particularly in response to unrest in various regions worldwide, notably in Africa and the Middle East. Consequently, many European nations like Sweden have become prominent desired destinations for numerous immigrants and refugees globally. The escalating political tension and unrest in regions such as the Middle East and Africa has also increased the immigrant flow to Europe. Subsequently, this has resulted in immigration becoming a hot topic among political parties in several European countries [15]including Sweden [16]where many immigrants are experiencing marginalization and discrimination. Thus, despite their initial potential health advantage (healthy immigrant effect) [17–20]they may become vulnerable to psychological distress leading to mental health disorders, such as mood disorders, even in young ages [21]. All these emerging trends highlight the need to understand the experiences of immigrant populations and families in host countries like Sweden and how such processes may impact their mental health. One of the measures of fair living conditions of the immigrants in their host country can be their comparable health effects of their economic and human capital when compared to non-immigrants (equal returns of equal resources) [22].

This retrospective European follow-up cohort study aims to compare immigrant and non-immigrant families in Sweden regarding the association between family SES indicators and subsequent incidence of mood disorders in youth. Grounded in the MDRs theory and conceptualizing immigration status as the source of social marginalization, we hypothesized weakened associations between family SES indicators and youth mood disorders for immigrant families compared to native-born families. As immigrants from African countries and the Middle East may bear visible social identities leading to additional discrimination [23]we hypothesized more pronounced diminished returns of SES for immigrant families from these regions compared to those from Europe [9].

## Materials and methods

We collected information on individuals from Swedish population-based registers with national coverage linking each person’s unique personal identity number which, to preserve confidentiality, was replaced with a serial number by Statistics Sweden [24]. We secured ethical approval for this study from the Swedish Ethical Review Authority (2021–04268). The Ethical Review Authority judged that individual consent was not needed. Our database consisted of all individuals residing for at least 12 months between ages 12 and 18 in Sweden at some point between Jan 1, 2001, and Dec 31, 2020. We included individuals between ages 12 to 18 years because these six years mark the last stage in compulsory school (when the teaching methods become more focused on independence) and high school. In addition, 18 years of age marks the start of adulthood when patients with psychiatric diseases will no longer be treated in child psychiatric care. The selected duration of study was based on data availability. Individuals were excluded if they lacked information on one or both parents in the Multigenerational register. In the database, we included information on the first registration of a mood disorder using ICD10 codes F32, F33, F40, F41, and F43 from outpatient visits and hospital admissions registered in the Swedish National Patient Register (NPR) [25]. In our definition of mood disorders we also included some neurotic disorders that are highly related to mood disturbances. For additional information on registers, see Appendix Table 1. Furthermore, we included information on immigrant status and three indicators of socioeconomic status (SES): parental education, parental employment status, family income) and family composition. Immigrant status was divided into four groups: natives (born in Sweden to Swedish-born parents), second-generation immigrants*1* (born in Sweden with *one* foreign-born parent), second-generation immigrants*2* (born in Sweden with *two* foreign-born parents), and first-generation immigrants (born outside Sweden with two foreign-born parents). The definitions of the included variables are presented in the Appendix Table 2.

To investigate the diminished returns theory, we used Cox proportional hazards models with time to mood disorder as outcome. We report the hazard ratio (HR) and 95% confidence intervals (CIs). In the models, individual observation started at age 12 years, Jan 1 2001, the date of immigration to Sweden (whichever came last), and ended at age 18, emigration date, date of death, Dec 31 2020, or date of first registration for mood disorder (whichever came first). We first performed separate analyses for the three family SES indicators and family composition (predictor variables). For each indictor we, in Model A1-4, included the predictor variable while controlling for sex, year of birth and immigrant status. In Model B we additionally included an interaction term between the predictor variable and immigrant status. We used this variable to investigate the theory of diminished returns. A positive interaction would suggest that the effect of the predictor variable is weaker for immigrant groups. Finally, we included all predictor variables and their interactions with immigrant status in the same model. In additional models, we divided the group born outside Sweden into 6 different groups based on the region of birth (Western countries, Middle East/North Africa, Africa (not North Africa), Asia (not the Middle East), Eastern Europe, Latin America) and applied the same analytic approach as described above. In all models, we tested the proportionality assumption [26, 27] by dividing the effect of the predictor variable into two groups: one effect for the ages 12–15 years and one effect for the ages 15–18 years. The Cox proportional hazards model assumes that hazard ratios remain constant over time. Testing the proportionality assumption is essential because violations can lead to biased estimates and incorrect conclusions. If the effect of a predictor variable changes over time, the model may misrepresent the true risk. Checking this assumption ensures the validity and interpretability of the results, especially in research of high relevance for policy or clinical implications. Finally, as a sensitivity analysis, we ran the main analyses (Cox regression models) using ordinary least squares (OLS) regression to determine whether the difference reflects a lower overall probability of diagnosis, rather than a later onset within the observation window. All analyses were performed using SAS 9.4.

## Results

### Descriptive data

As shown in Table 1, with the exception of living with both parents, all SES indicators, namely parental education, family income, and parental employment, were highest for natives and lowest for first-generation immigrants. Second-generation immigrants fell in between the two groups mentioned above. Living with both parents was highest for first-generation immigrants and lowest for natives.

In our analysis, a total of 2,295,738 individuals who resided in Sweden at some point between the ages of 12 and 18 were included. Among them, 1,696,047 (73.9%) were native, 225,779 (9.8%) were Swedish-born with one foreign-born parent (second-generation immigrant*1*), 192,908 (8.4%) were Swedish-born with both parents foreign-born (second-generation immigrant*2*), and 181,004 (7.9%) were born outside Sweden (first-generation immigrant). The incidence rates of mood disorders were 7.0, 7.4, 8.9, 4.5, and 3.9 per 1,000 person-years in these groups, respectively.

**Table 1:** Characteristics of study participants 12–18 years stratified by immigration background in Sweden 2001–2020

	Total (*n* = 2,295,738)	Native (*n* = 1,696,047)	Swedish born		Foreign-born (*n* = 181,004)
			One foreign-born parent (*n* = 225,779)	Two foreign-born parents (*n* = 192,908)	
Sex					
Male	51.7%	51.6%	51.5%	51.4%	52.9%
Female	48.4%	48.4%	48.5%	48.6%	47.2%
Year of birth, mean (SD)	1994 (6.3)	1994 (6.2)	1995 (6.3)	1996 (6.0)	1996 (6.5)
Education					
Low Education	31.1%	29.9%	29.8%	42.1%	36.7%
Mid Education	20.4%	20.2%	19.3%	20.3%	24.6%
High Education	48.2%	50.0%	50.8%	37.6%	38.7%
Residing with both parents					
Yes	71.1%	71.0%	59.6%	69.5%	87.1%
No	29.0%	29.0%	40.4%	30.5%	12.9%
Parental Employment					
Both parents working	49.4%	55.4%	46.2%	32.2%	15.0%
One parent working	13.4%	11.1%	18.0%	22.1%	20.8%
None	37.2%	33.6%	35.8%	45.7%	64.2%
Income					
Low	25.0%	20.7%	29.3%	31.7%	53.6%
Mid-Low	25.0%	24.3%	23.7%	29.3%	28.7%
Mid-High	25.0%	26.8%	23.0%	23.3%	11.9%
High	25.0%	28.2%	23.9%	15.7%	5.9%
Missing (N)	21,814	9,297	2,208	1,816	8,493
Mood disorder diagnosis	4.5%	4.7%	5.9%	3.3%	2.4%

### Intersectional data

Table 2 presents incidence rates of mood disorders per 1,000 person-years, both overall (i.e., the entire study sample) and stratified by immigration background and key family-level socioeconomic indicators, and family composition. As shown in this table, overall (7.7 vs. 7.3 vs. 6.3 per 1,000 person-years, respectively) and for native children (8.8 vs. 7.7 vs. 6.5 per 1,000 person-years, respectively), there was a gradual decrease in the risk of mood disorders as parental education levels went from low to mid to high. This pattern was not consistently observed, with a different trend for Swedish-born individuals with foreign-born parents (4.3 vs. 4.2 vs. 4.6 per 1,000 person-years, respectively) and for children born outside Sweden (3.5 vs. 4.7 vs. 3.7 per 1,000 person-years, respectively).

Similarly, overall (9.3 vs. 7.0 vs. 6.3 vs. 5.1 per 1,000 person-years, respectively) and native children (11.1 vs. 8.0 vs. 6.8 vs. 5.0 per 1,000 person-years, respectively), showed a stepwise decline in the risk of mood disorders with increasing income levels. However, this stepwise declining trend was inconsistent for Swedish-born children/youth with foreign-born parents (5.6 vs. 4.2, 3.5 vs. 2.9 per 1,000 person-years, respectively) and children/youth born outside Sweden (4.0 vs. 3.1 vs. 2.6 vs. 3.0 per 1,000 person-years, respectively).

**Table 2:** The incidence of mood disorders per 1000 person-years, overall and by intersection of nativity and family socioeconomic status (SES) and family composition

	Total (*n* = 2,295,738)	Native (*n* = 1,696,047)	Swedish born		Foreign-born (*n* = 181,004)
			One foreign- born parent (*n* = 225,779)	Two foreign-born parents (*n* = 192,908)	
Parental Education					
Low Education	7.7 (7.6; 7.8)	8.8 (8.7; 8.9)	10.5 (10.1; 10.8)	4.3 (4.1; 4.5)	3.5 (3.3; 3.7)
Mid Education	7.3 (7.2; 7.4)	7.7 (7.5; 7.8)	9.0 (8.7; 9.4)	4.2 (3.9; 4.4)	4.7 (4.4; 5.0)
High Education	6.3 (6.3; 6.4)	6.5 (6.4; 6.6)	8.0 (7.8; 8.2)	4.6 (4.4; 4.8)	3.7 (3.6; 3.9)
Residing with both parents					
Yes	5.5 (5.4; 5,5)	5.8 (5.8; 5.9)	6.6 (6.5; 6.8)	3.5 (3.3; 3.6)	3.6 (3.5; 3.8)
No	10.8 (10.6; 10.9)	11.3 (11.2; 11.4)	12.4 (12.2; 12.7)	6.7 (6.5; 7.0)	5.6 (5.2; 6.0)
Parental Employment					
Both parents working	6.0 (5.9; 6.1)	6.1 (6.0; 6.2)	7.0 (6.8; 7.2)	3.4 (3.3; 3.6)	3.4 (3.1; 3.7)
One parent working	8.4 (8.2; 8.7)	10.1 (9.8; 10.4)	9.9 (9.4; 10.4)	4.6 (4.3; 5.0)	3.5 (3.1; 4.0)
None	8.2 (8.0; 8.4)	10.7 (10.5; 11.0)	10.2 (9.6; 10.8)	3.8 (3.5; 4.1)	2.8 (2.6; 3.0)
Family Income					
Low	9.3 (9.2; 9.4)	11.1 (11.0; 11.3)	11.9 (11.5; 12.2)	5.6 (5.4; 5.8)	4.0 (3.9; 4.2)
Mid-Low	7.0 (6.9; 7.1)	8.0 (7.8; 8.1)	9.3 (9.0; 9.6)	4.2 (4.1; 4.5)	3.1 (2.9; 3.3)
Mid-High	6.3 (6.2; 6.3)	6.8 (6.7; 6.9)	7.7 (7.4; 8.0)	3.5 (3.3; 3.7)	2.6 (2.4; 2.8)
High	5.1 (5.0; 5.1)	5.0 (4.9; 5.1)	5.7 (5.5; 6.0)	2.9 (2.7; 3.1)	3.0 (2.6; 3.3)
Mood disorder diagnosis	7.0 (6.9;7.0)	7.4 (7.3; 7.5)	8.9 (8.8; 9.1)	4.5 (4.4; 4.6)	3.9 (3.8; 4.1)

The incidence of mood disorders per 1000 person-years, overall and by intersection of nativity and family socioeconomic status (SES) and family composition

### Overall cox regressions

Table 3 presents results from a series of models—beginning with main effects (earlier models) and progressing to models that include interaction terms (later models). Across all models, findings suggest that the protective effects of socioeconomic resources—particularly income and education—are not equally distributed across population subgroups supporting the MDR theory.

#### Models 1 A and 1B: base models (adjusted for sex, year of birth, and immigrant background)

Models 1 A and 1B assess the association between immigrant background and the risk of mood disorders, adjusting for sex and year of birth. Compared to native-born youth (reference group), group 2 (Swedish-born with one foreign-born parent) showed elevated risk: HR = 1.21 (95% CI: 1.19–1.23) in Model 1 A and HR = 1.19 (95% CI: 1.15–1.23) in Model 1B. In contrast, both Group 3 (Swedish-born with two foreign-born parents) and Group 4 (foreign-born youth) showed lower risk: Group 3 h = 0.58 (95% CI: 0.56–0.59) in 1 A and HR = 0.48 (95% CI: 0.46–0.50) in 1B; Group 4 h = 0.49 (95% CI: 0.48–0.51) in 1 A and HR = 0.38 (95% CI: 0.36–0.41) in 1B. Males had consistently lower risks compared to females (HR = 0.43; 95% CI: 0.43–0.44), and the risk slightly increased in more recent birth years (HR = 1.11; 95% CI: 1.11–1.12 in both models).

#### Models 2 A and 2B: addition of family composition

When family composition was included, hazard ratios for immigrant groups were modestly attenuated. For Group 2, HRs declined to 1.12 (95% CI: 1.10–1.14) in Model 2 A and 1.10 (95% CI: 1.08–1.13) in Model 2B. Group 4 showed slightly increased HRs: 0.58 (95% CI: 0.56–0.60) in 2 A and 0.49 (95% CI: 0.45–0.52) in 2B. Living with both parents was associated with significantly lower risk: HR = 0.52 (95% CI: 0.51–0.52) in Model 2 A and HR = 0.51 (95% CI: 0.51–0.52) in Model 2B.

#### Models 3 A and 3B: addition of parental employment

These models introduced parental employment status. Compared to youth with no employed parents (reference), those with both parents employed had reduced risk of mood disorders: HR = 0.76 (95% CI: 0.75–0.77) in Model 3 A and HR = 0.78 (95% CI: 0.77–0.79) in Model 3B. In contrast, having only one employed parent was associated with elevated risk: HR = 1.19 (95% CI: 1.17–1.22) in 3 A and HR = 1.35 (95% CI: 1.32–1.38) in 3B. Interaction terms revealed variation by immigrant background. For Group 4, the protective effect of both parents working was reduced or even reversed: HR = 1.15 (95% CI: 1.05–1.25) in Model 3B. For Group 3, having one employed parent was protective: HR = 0.62 (95% CI: 0.58–0.67).

#### Models 4 A and 4B: addition of family income

In models that included family income, a strong protective gradient emerged. Compared to youth from low-income households (reference), those from high-income households had substantially lower risk: HR = 0.46 (95% CI: 0.45–0.47) in Model 4 A and HR = 0.45 (95% CI: 0.44–0.46) in Model 4B. Protective effects were also observed for mid-low and mid-high-income levels. However, income × immigrant background interaction terms revealed diminished returns. Among Group 4 youth, those in high-income households experienced higher risk: HR = 1.69 (95% CI: 1.50–1.92) in Model 4B. A similar, though weaker, pattern appeared for Group 3: HR = 1.15 (95% CI: 1.07–1.25), indicating attenuated or even reversed protective effects of income.

#### Models 5 A and 5B: full model Including parental education

In the final models, parental education was reintroduced alongside family income, employment, and composition. Among native youth, higher parental education remained protective: HR = 0.92 (95% CI: 0.90–0.93) for high education in Model 5 A. However, interaction terms again pointed to diminished returns among immigrant groups. For Group 3, high parental education was associated with increased risk: HR = 1.35 (95% CI: 1.28–1.44); for Group 4, HR = 1.27 (95% CI: 1.18–1.36), both in Model 5B. Income interaction effects persisted but were slightly attenuated. For example, the HR for Group 4 with high income declined from 1.69 in Model 4B to 1.11 (95% CI: 0.97–1.27) in Model 5B, suggesting some adjustment but continued evidence of diminished returns. According to our models in Table 3, SES indicators—parental employment, family income, and parental education, as well as having 2-parent family—were generally protective against mood disorders. However, interaction terms consistently revealed that the magnitude of these protective effects was weaker for immigrant youth. These findings provide empirical support for the MDRs framework, indicating that socioeconomic advantages do not translate equally across social groups, particularly for those with immigrant backgrounds.1

**Table 3:** Cox regression model with time to registration of mood disorder. The Hazard Ratio (HR) for the effect of family socioeconomic status (SES) and family composition across different immigrant groups

	Model1 A	Model1 B	Model2 A	Model2 B	Model3 A	Model3 B	Model4 A	Model4 B	Model5 A	Model5 B	
Sex											
Females	Reference	Reference	Reference	Reference	Reference	Reference	Reference	Reference	Reference	Reference	
Males	0.43 (0.43; 0.44)	0.43 (0.42; 0.44)	0.43 (0.43; 0.44)	0.43 (0.43; 0.44)	0.43 (0.43; 0.44)	0.43 (0.43; 0.44)	0.43 (0.42; 0.43)	0.43 (0.42; 0.44)	0.43 (0.42; 0.43)	0.43 (0.42; 0.43)	
Year of Birth	1.11 (1.11; 1.12)	1.11 (1.11; 1.12)	1.11 (1.11; 1.12)	1.11 (1.11; 1.11)	1.12 (1.12; 1.12)	1.12 (1.12; 1.12)	1.13 (1.13; 1.13)	1.13 (1.13; 1.13)	1.13 (1.13; 1.13)	1.13 (1.13; 1.13)	
Immigrant background											
Natives	Reference	Reference	Reference	Reference	Reference	Reference	Reference	Reference	Reference	Reference	
Group 2 (G2)	1.21 (1.19; 1.23)	1.19 (1.15; 1.23)	1.12 (1.10; 1.14)	1.10 (1.08; 1.13)	1.17 (1.14; 1.19)	1.28 (1.24; 1.32)	1.12 (1.10; 1.14)	1.07 (1.03; 1.10)	1.09 (1.07; 1.11)	1.12 (1.07; 1.18)	
Group 3 (G3)	0.58 (0.56; 0.59)	0.48 (0.46, 0.50)	0.59 (0.58; 0.61)	0.59 (0.56; 0.61)	0.55 (0.54; 0.56)	0.63 (0.61; 0.66)	0.52 (0.41; 0.53)	0.50 (0.48; 0.52)	0.53 (0.52; 0.54)	0.53 (0.50; 0.57)	
Group 4 (G4)	0.49 (0.48; 0.51)	0.38 (0.36; 0.41)	0.58 (0.56; 0.60)	0.49 (0.45; 0.52)	0.44 (0.43; 0.46)	0.49 (0.47; 0.51)	0.37 (0.36; 0.38)	0.36 (0.35; 0.38)	0.45 (0.43; 0.46)	0.42 (0.38; 0.46)	
Residing with Both parents											
No	-	-	Reference	Reference	-	-	-	-	Reference	Reference	
Yes	-	-	0.52 (0.51; 0.52)	0.51 (0.51; 0.52)	-	-	-	-	0.61 (0.60; 0.61)	0.61 (0.60; 0.62)	
Parental education											
Low	Reference	Reference	-	-	-	-	-	-	Reference	Reference	
Mid	0.90 (0.88; 0.91)	0.87 (0.85; 0.89)	-	-	-	-	-	-	0.95 (0.93; 0.97)	0.93 (0.91; 0.95)	
High	0.77 (0.76; 0.78)	0.74 (0.72; 0.75)	-	-	-	-	-	-	0.92 (0.90; 0.93)	0.88 (0.87; 0.90)	
Parental Employment											
None	-	-	-	-	Reference	Reference	-	-	Reference	Reference	
One	-	-	-	-	1.19 (1.17; 1.22)	1.35 (1.32; 1.38)	-	-	1.06 (1.04; 1.08)	1.15 (1.12; 1.17)	
Both	-	-	-	-	0.76 (0.75; 0.77)	0.78 (0.77; 0.79)	-	-	0.84 (0.83; 0.85)	0.85 (0.84; 0.87)	
Family Income											
Low	-	-	-	-	-	-	Reference	Reference	Reference	Reference	
Mid-Low	-	-	-	-	-	-	0.74 (0.72; 0.75)	0.72 (0.71; 0.74)	0.90 (0.88; 0.92)	0.91 (0.89; 0.93)	
Mid-High	-	-	-	-	-	-	0.61 (0.60; 0.62)	0.60 (0.59; 0.61)	0.86 (0.84; 0.88)	0.87 (0.85; 0.89)	
High	-	-	-	-	-	-	0.46 (0.45; 0.47)	0.45 (0.44; 0.46)	0.74 (0.72; 0.76)	0.75 (0.73; 0.76)	
G2* Mid Education		0.99 (0.94; 1.05)	-	-	-	-	-	-	-	1.00 (0.95; 1.05)	
G2* High Education		1.03 (0.99; 1.08)	-	-	-	-	-	-	-	1.02 (0.98; 1.08)	
G3* Mid Education	-	1.12 (1.04; 1.20)	-	-	-	-	-	-	-	1.11 (1.04; 1.20)	
G3* High Education	-	1.46 (1.39; 1.56)	-	-	-	-	-	-	-	1.35 (1.28; 1.44)	
G4 * Mid Education	-	1.53 (1.41; 1.66)	-	-	-	-	-	-	-	1.44 (1.33; 1.57)	
G4 * High Education	-	1.46 (1.37; 1.58)	-	-	-	-	-	-	-	1.27 (1.18; 1.36)	
G2 * Both parents	-	-	-	1.03 (1.00; 1.07)	-	-	-	-	-	1.00 (0.96; 1.05)	
G3 * Both parents	-	-	-	1.00 (0.95; 1.05)	-	-	-	-	-	0.91 (0.86; 0.97)	
G4 * Both parents	-	-	-	1.23 (1.15, 1.34)	-	-	-	-	-	1.04 (0.96; 1.13)	
G2* One parent working	-	-	-	-	-	0.75 (0.71; 0.79)	-	-	-	0.80 (0.76; 0.84)	
G2* Both parents working	-	-	-	-	-	0.90 (0.87; 0.94)	-	-	-	0.94 (0.90; 0.98)	
G3* One parent working	-	-	-	-	-	0.62 (0.58; 0.67)	-	-	-	0.70 (0.65; 0.75)	
G3* Both parents working	-	-	-	-	-	0.89 (0.84; 0.94)	-	-	-	0.90 (0.84; 0.96)	
G4 * One parent working	-	-	-	-	-	0.57 (0.52; 0.61)	-	-	-	0.67 (0.62; 0.73)	
G4 * Both parents working	-	-	-	-	-	1.15 (1.05; 1.25)	-	-	-	1.17 (1.06; 1.29)	
G2* Mid-Low Income	-	-	-	-	-	-	-	1.09 (1.04; 1.15)	-	1.04 (0.99; 1.10)	
G2* Mid-High Income	-	-	-	-	-	-	-	1.07 (1.01; 1.12)	-	1.02 (0.96; 1.18)	
G2* High Income	-	-	-	-	-	-	-	1.08 (1.03; 1.13)	-	1.04 (0.98; 1.11)	
G3* Mid-Low Income	-	-	-	-	-	-	-	1.04 (0.98; 1.11)	-	1.01 (0.95; 1.08)	
G3* Mid-High Income	-	-	-	-	-	-	-	1.02 (0.95; 1.10)	-	1.00 (0.92; 1.08)	
G3* High Income	-	-	-	-	-	-	-	1.15 (1.07; 1.25)	-	1.06 (0.97; 1.17)	
G4 * Mid-Low Income	-	-	-	-	-	-	-	1.04 (0.96; 1.10)	-	0.89 (0.83; 0.96)	
G4 * Mid-High Income	-	-	-	-	-	-	-	1.07 (0.97; 1.18)	-	0.81 (0.73; 0.91)	
G4 * High Income	-	-	-	-	-	-	-	1.69 (1.50; 1.92)	-	1.11 (0.97; 1.27)	

Group 1 (G1) Native; Group 2 (G2) Swedish-born with 1 foreign-born parent; Group 3 (G3) Swedish-born with foreign-born parents) Group 4 (G4) Born outside Sweden

As shown in Table 4, for first generation immigrants, we found interactions between middle-level parental education and three regions namely Middle East/North Africa, Africa, and Asia. These interactions were indicative of weaker protective effects of parental education (mid-level) on subsequent mood disorders of youth for immigrants from these regions not families immigrated from East of West Europe. We did not find a similar pattern for other SES indicators.

**Table 4:** Cox regression model with time to registration of mood disorder. Hazard ratio (HR) of effects of highest parental education and region of origin of immigrants

	Model A	Model B
Highest parental education		
Females	Reference	Reference
Males	0.49 (0.46; 0.53)	0.49 (0.46; 0.53)
Year of Birth	1.06 (1.06; 1.07)	1.06 (1.06; 1.07)
Region or origin		
Western Europe/North America/Australia (West)	Reference	Reference
Middle East/North Africa	0.66 (0.60; 0.73)	0.66 (0.55; 0.80)
Africa (not North Africa)	0.35 (0.30; 0.40)	0.29 (0.23; 0.37)
Asia (not Middle East)	0.86 (0.76; 0.96)	0.78 (0.63; 0.97)
Eastern Europe	0.70 (0.63; 0.78)	0.69 (0.56; 0.85)
Latin America	0.97 (0.79; 1.19)	0.86 (0.57; 1.28)
Parental education		
Low Education	Reference	Reference
Mid Education	1.14 (1.05; 1.23)	0.77 (0.60; 1.00)
High Education	0.95 (0.89; 1.02)	1.03 (0.85; 1.26)
Interaction of region or origin*education		
Middle East/North Africa * Mid Education	-	1.57 (1.18; 2.09)
Middle East/North Africa * High Education	-	0.82 (0.65; 1.03)
Africa (not North Africa) * Mid Education	-	2.08 (1.42; 3.04)
Africa (not North Africa) * High Education	-	1.11 (0.78; 1.59)
Asia (not Middle East) * Mid Education	-	2.01 (1.45; 2.77)
Asia (not Middle East) * High Education	-	0.83 (0.62; 1.10)
Eastern Europe * Mid Education	-	1.25 (0.92; 1.69)
Eastern Europe * High Education	-	0.99 (0.77; 1.28)
Latin America * Mid Education	-	1.05 (0.54; 2.03)
Latin America * High Education	-	1.22 (0.76; 1.98)

### Sensitivity analysis

We repeated our main analysis using OLS (Appendix Tables 1, 2, 3, 4 and 5). First, the results showed that individuals with two foreign-born parents, as well as individuals born outside Sweden, had lower rates of mood disorders even after controlling for all covariates. Second, the analysis illustrated the interaction on an additive scale rather than a multiplicative scale. The additive interaction results were broadly consistent with those from the multiplicative model, indicating that while the protective effects remained, their magnitude was weaker for immigrant youth.

## Discussion

Our study had three important findings. First, all family SES indicators were protective against future risk of youth mood disorders. These protective effects could be observed for parental education, parental employment, family income, and both parents being present in the household. Our second finding showed that, at least for parental education and employment, these protective effects were strongest for native-born youth and weakest for foreign-born youth with foreign-born parents. Diminished returns of these two-family SES indicators on youth mood disorders were confirmed by statistically significant interactions between immigration status and parental education and employment. According to this second finding, youth mood disorder does not seem to follow a similar social gradient for mood disorders resulting from parental education and employment in immigrants compared to non-immigrant youth in Sweden. Our third finding partially confirmed our hypothesis that immigrants from Africa, North Africa/Middle East, and Asia but not immigrants from Western and Eastern Europe had weaker protective effects of mid-level parental education. Multiple explanations can be proposed for the weaker effects of education on outcomes of immigrants. One, although only suggestive, more pronounced diminished returns for parental education can be due to racism because these diminished returns were most pronounced for immigrants from countries with more visible or apparent marginalizing identities based on skin color, race, or religion. Other factors contributing to these disparities might include the hiring preferences and practices of employers, which may not necessarily be driven by overt racism. For instance, employers might prioritize candidates who they perceive as fitting into the company culture easily and minimizing potential risks, rather than intentionally discriminating based on race. This inclination to hire individuals who align with the existing work group’s dynamics, though not explicitly racist, can inadvertently disadvantage marginalized groups. Another factor contributing to this phenomenon is the origin of education. For immigrants whose education was obtained outside of Sweden, there is often a need for validation or adaptation to Swedish standards, rendering the education less immediately applicable compared to those with education from within Sweden. Education obtained in Sweden may be more readily recognized and preferred by Swedish employers, thereby yielding greater benefits and outcomes for individuals who have pursued education within the country. Difficulties to capitalize on one’s education in combination with the lowered status in the new country can create additional frustration and poor psychological well-being.

Regarding our first finding, a large body of research by Braveman [2, 28, 29, 30, 31, 32, 33, 34]and others has established a connection between SES and health. For example, fundamental cause (i.e., social resources influence multiple disease outcomes that cause affects disease outcomes through multiple risk factors) [1, 2, 35]social determinants [36]and social gradient [37] frameworks posit that there is a positive association between SES indicators and health; populations, families and individuals with higher SES resources tend to experience better health [38]. Many original studies have also documented the enduring importance of various SES indicators namely income, education, employment, and number of parents in the household as major contributors to human health [39, 40].

Regarding our second finding, this is not the first study to show weaker association between family or individual SES and health outcomes in immigrant populations\ [41–46]. It is, however, the first follow-up study in Europe that documents similar results as in the US, where cross-sectional studies have shown that education has larger positive effects on psychological distress, self-rated health, and chronic diseases in non-immigrant populations compared to immigrant populations [5, 22].

In connection to our third observation, we partially confirmed our hypothesis that immigrants from Africa, North Africa/Middle East, and Asia had a comparatively weaker protective influence of parental (mid-level) education. Importantly, this diminished effect was not observed among immigrants from Western and Eastern Europe. This observation implies that the most pronounced reduction in the protective impact of parental education was evident in countries of origin marked by more prominent marginalizing identities linked to factors such as skin color, religion, or race. However, it’s important to note that this finding remains suggestive, lacking replication across other SES indicators, which partly diminishes its overall robustness. This finding is, however, in support of results by Bakhtiari on self-rated health as the outcome [9].

Although the primary focus of this study was not on the main effects of immigration, an interesting observation that emerged was the reduced risk of mood disorders among immigrant youth compared to their native-born counterparts. The incidence of mood disorders in the youths was 7.4, 8.9, 4.5, and 3.9 per 1,000 person-years in native-born, Swedish-born with one foreign-born parent, Swedish-born with two foreign-born parents, and foreign-born with foreign-born parents, respectively. This observation aligns with the well-documented healthy immigrant effect [17–19]a phenomenon widely recognized in research in Europe and US. It also resonates with the concept of heightened resilience among populations facing adversity, a phenomenon observed in black populations in the U.S., where they, despite their lower SES, exhibit a lower prevalence of mood disorders compared to Whites. This means that, similar to the Black mental health paradox [47] in the US, there might exist an immigrants’ mental health paradox [19, 48]in Europe or at least in Sweden.

It has been suggested that the function and structure of society is responsible for diminished returns of SES for marginalized populations. Research shows that the US social institutions and people differentially treat people based on their nationality, heritage, ethnicity, race, color, and social class [49],. Unfortunately, all marginalized groups seem to receive worse treatment and are discriminated [50]. The observation that diminished returns of SES hold for all marginalized groups suggests that these patterns are robust and consistent. This means that any deviation from belonging to the majority group, i.e., US-born non-Latino Whites will be associated with a penalty, which could show its effect with weaker than expected effects of education and human capital on health outcomes in socially marginalized populations.

In the US, systemic marginalization of any non-majority social group has been observed. Social exclusion and marginalization may reduce individuals’ chances of full participation in social activities and of taking full advantage of offered opportunities. As such, even highly educated immigrants do not seem to enjoy the benefits from their human capital. Similar to racism that reduces the health returns of education for Black and Latino people, xenophobia and toxic nationalism may reduce immigrant individuals’ ability to leverage their human capital and secure tangible outcomes such as a good health. Other explanatory models consider how well education is adapted to Swedish conditions and the overall integration into Swedish working life. These models suggest that success in the job market may not solely depend on having the right education but also on how well an individual is integrated into the Swedish work culture. These differences are not necessarily rooted in racism but rather in the ability to effectively navigate and contribute to Swedish working life. As a result, the expected benefits that should follow education would be reduced [5, 22].

Diminished returns of human and material resources are not limited to education [51] and can be observed for many other resources and assets such as income [52]occupation [53]and marital status [54]. All these resources show weaker health effects for marginalized than privileged groups. Similarly, these diminished returns are not only observed for self-rated health [51, 55, 56] but also for psychological distress [56]obesity [10, 57]chronic diseases [58, 59]hospitalization [60]and mortality [53]. Similarly, highly educated marginalized people are more likely to vape [61]smoke [62–66]drink [67, 68] and have a poor diet [69] and less exercise [70]. Thus, these diminished returns are neither specific to a particular resource nor a particular outcome.

Our study represents the first longitudinal exploration of MDRs among immigrants in Europe. The observed MDRs for immigrants in Europe align with well-established cross-sectional literature in the US and emerging research in Europe. In a European context, Bakhtiari’s research, encompassing surveys from 30 countries over six waves of the European Social Survey (ESS), demonstrated the interaction between SES and immigration/ethno-racial minority status. The findings revealed a diminished returns pattern, particularly affecting populations with origins in Sub-Saharan Africa and the Middle East. The authors emphasized the significance of cross-national comparisons in understanding variations in how racism and SES interact to influence health disparities. In the US, established literature highlights diminished SES effects on several health indicators. For instance, an analysis of the ABCD study, including 9–10-year-old children, revealed weaker effects of family SES on the executive function of immigrant families compared to native-born families. Similarly, studies involving adults consistently demonstrate the nuanced relationship between SES and health outcomes.

The observed diminished effect of education is in line with a study using the National Health Interview Survey (NHIS), showing that the effects of education on psychological distress, self-rated health, and chronic diseases were weaker for immigrants than non-immigrants [45].

The diminished returns of family SES indicate that additional family SES might not effectively shield immigrant families from experiences of discrimination, precarious work conditions, and limited access to quality healthcare [48]. It is therefore crucial for clinicians and healthcare providers to be aware that immigrants, irrespective of their family SES levels, may encounter structural barriers due to immigration laws, labor market regulations, and residential segregation. These barriers potentially undermine the health benefits linked to upward social mobility among immigrants.

It is possible that education, when acquired outside the host country, may have weaker effects on changing the living conditions of the individuals in the host country. This notion implies some degrees of explainable diminishing returns for education, possibly due to variations in the quality of education (a part of diminished returns that might be fair). This argument, however, only pertains to education and cannot justify reduced health returns of employment, income, and family composition in immigrant families. The systemic nature of the observed diminished returns (seen for all SES indicators) above and beyond education suggests that structural or cultural factors such as marginalization and stigmatization of immigrants may be at play. This invites future research on structural mechanisms that can hinder the lives of immigrants regardless of their achievements. A recent study showed that xenophobia at the country level is associated with reduced happiness in immigrants in Europe [71].

For at least four reasons, we believe that our results of diminished returns of SES on mood disorders among immigrant children/youth are robust: First, the results held across all included SES indicators. Second, the strongest diminished returns were observed in the most disadvantaged groups (first-generation immigrants) and middle education for second-generation immigrants, suggesting that there might be a dose-dependent effect of immigration on diminished returns. Third, our sensitivity analyses consistently supported our main findings. Fourth, we did not find any differences in our observed patterns for males vs. females. This evidence collectively suggests that the observed MDRs are not statistical artifacts or a result of a regression towards the mean [72].

A remaining question is to what degree these results are influenced by actual need or utilization of mental health services [73, 74]. If our results are related to lower utilization of services, a higher prevalence should be viewed as positive, indicating that the healthcare need is addressed, and that people are receiving the care they need.

### Limitations

This study is subject to a few methodological limitations. Firstly, the absence of data on wealth, home ownership, and acculturation (e.g., language knowledge) limited our study. We could also not decompose the role of the need for healthcare vs. utilization of healthcare in our analysis. Our reported rates may, therefore, be reflective of either need or utilization. Future studies could include more detailed analyses, based on interviews and survey data, to explore whether our findings could be related to cultural barriers to seek health care or psychiatric ill health among the parents. Furthermore, the study did not encompass more common elements of mood disorders [75]such as those not leading to a clinical diagnosis. Moreover, the decision to treat SES as fixed (at baseline) while hypothesizing that parental education is relatively stable raises concerns. Family income and employment status may improve in the years following integration and immigration. To address this, a sensitivity analysis was conducted three years later to test if the results held, considering the increased time for integration and potential improvements in SES. Our findings remained consistent, indicating that this limitation had minimal impact on our study. Although we interpret the weaker protective effects of family SES on mood disorders among immigrant families in contrast to natives as potentially reflecting diminished returns of human and material resources, this interpretation should be approached with caution. Our study did not account for the country in which parents obtained their education or whether immigrants’ educational credentials were recognized or devalued in the Swedish context. These unmeasured factors may contribute to the observed patterns and could offer alternative explanations. Future research should directly examine the role of credential recognition and the structural devaluation of immigrant education on immigrant youth mood disorders. This limitation should be considered when interpreting our findings and warrants further discussion. Finally, we did not have data on race/ethnicity as our information was limited to the country of origin. Despite these limitations, this study has several strengths, representing the first longitudinal examination of the diminishing returns of SES over time in a substantially large birth cohort in Sweden.

## Conclusion

Our study findings support weaker protective effects of family SES indicators on mood disorders among youth in immigrant families, in contrast to non-immigrant families in Sweden. To address the mental health needs of immigrant youth, it is essential to implement two types of policies. The first set of policies may aim to support education and employment in general in immigrant families. The second set of required policy interventions may address structural barriers that hinder immigrant families from securing tangible health outcomes in their host countries when they climb the social ladder. While the first set of policies may help immigrant families gain financial, human, and material resources, the second set of policies may help them leverage their existing human capital in the host countries. This is especially important for immigrants originating from Africa and the Middle East, who may have more visible identities that could more easily lead to marginalization. In summary, to equalize the health returns of family SES and family composition for immigrant and non-immigrant families, underlying societal and structural processes should be examined further in future studies.

## Supplementary Information

Supplementary Material 1.

## Data Availability

Based on Swedish law, the register data (used in this study) could not be shared publicly.

## References

[1] Link BG, Phelan J. Social conditions as fundamental causes of disease. J Health Soc Behav. 1995;Spec No:80–94. 7560851

[2] Phelan JC, Link BG. Fundamental Cause Theory. In: Cockerham, W. (eds) Medical Sociology on the Move. Dordrecht: Springer; 2013. 10.1007/978-94-007-6193-3.

[3] Reiss F. Socioeconomic inequalities and mental health problems in children and adolescents: a systematic review. Soc Sci Med. 2013;90:24–31. 23746605 10.1016/j.socscimed.2013.04.026

[4] Letourneau NL, Duffett-Leger L, Levac L, Watson B, Young-Morris C. Socioeconomic status and child development: a meta-analysis. J Emotional Behav Disord. 2013;21(3):211–24.

[5] Assari S. Health disparities due to diminished return among black Americans: public policy solutions. Soc Issues Policy Rev. 2018;12(1):112–45.

[6] Farmer MM, Ferraro KF. Are racial disparities in health conditional on socioeconomic status? Soc Sci Med. 2005;60(1):191–204. 15482878 10.1016/j.socscimed.2004.04.026

[7] Fuller-Rowell TE, Curtis DS, Doan SN, Coe CL. Racial disparities in the health benefits of educational attainment: a study of inflammatory trajectories among African American and white adults. Psychosom Med. 2015;77(1):33–40. 25490696 10.1097/PSY.0000000000000128

[8] Hudson DL, Bullard KM, Neighbors HW, Geronimus AT, Yang J, Jackson JS. Are benefits conferred with greater socioeconomic position undermined by racial discrimination among African American men? J Mens Health. 2012;9(2):127–36. 22707995 10.1016/j.jomh.2012.03.006 PMC3371660

[9] Bakhtiari E. Diminished returns in Europe: socioeconomic status and ethno-racial health disparities across 30 countries in the European social survey. J Racial Ethn Health Disparities. 2022;9(6):2412–26. 35094375 10.1007/s40615-021-01178-2

[10] Assari S, Thomas A, Caldwell CH, Mincy RB. Blacks’ diminished health return of family structure and socioeconomic status; 15 years of follow-up of a national urban sample of youth. J Urban Health. 2018;95(1):21–35. 29230628 10.1007/s11524-017-0217-3 PMC5862702

[11] Assari S, Caldwell CH. High risk of depression in High-income African American boys. J Racial Ethn Health Disparities. 2018;5(4):808–19. 28842841 10.1007/s40615-017-0426-1 PMC6556394

[12] Assari S, Boyce S, Caldwell CH, Bazargan M. Parent education and future transition to cigarette smoking: latinos’ diminished returns. Front Pediatr. 2020;8: 457. 32974240 10.3389/fped.2020.00457 PMC7466764

[13] Assari S, Boyce S, Bazargan M, Caldwell CH. Mathematical performance of American youth: diminished returns of educational attainment of Asian-American parents. Educ Sci. 2020. 10.3390/educsci10020032. PMC7083587 32201681

[14] Assari S, American Indian A, Native. Native hawaiian, and Pacific Islander children’s body mass index: diminished returns of parental education and family income. Res Health Sci. 2020;5(1):64–84. 34308086 10.22158/rhs.v5n1p64

[15] Kyriazi A, Mendes MS, Rone J, Weisskircher M. The politics of emigration in Europe: a research agenda. JCMS J Common Market Stud. 2023;61(2):563–75.

[16] Hellström A. The populist divide in far-Right political discourse in Sweden: anti-immigration claims in the Swedish socially conservative online newspaper Samtiden from 2016 to 2022. Societies. 2023;13(5):108.

[17] Constant AF, Garcia-Munoz T, Neuman S, Neuman T. A healthy immigrant effect or a sick immigrant effect? Selection and policies matter. Eur J Health Econ. 2018;19(1):103–21. 28144758 10.1007/s10198-017-0870-1

[18] Corlin L, Woodin M, Thanikachalam M, Lowe L, Brugge D. Evidence for the healthy immigrant effect in older Chinese immigrants: a cross-sectional study. BMC Public Health. 2014;14:603. 24928348 10.1186/1471-2458-14-603 PMC4067103

[19] Markides KS, Rote S. The healthy immigrant effect and aging in the united States and other western countries. Gerontologist. 2019;59(2):205–14. 30383212 10.1093/geront/gny136

[20] McDonald JT, Kennedy S. Insights into the ‘healthy immigrant effect’: health status and health service use of immigrants to Canada. Soc Sci Med. 2004;59(8):1613–27. 15279920 10.1016/j.socscimed.2004.02.004

[21] Rodriguez J, Radjack R, Moro MR, Lachal J. Migrant adolescents’ experience of depression as they, their parents, and their health-care professionals describe it: a systematic review and qualitative meta-synthesis. Eur Child Adolesc Psychiatry. 2024;33(1):1–19. 35301589 10.1007/s00787-022-01971-2

[22] Assari S. Unequal gain of equal resources across racial groups. Int J Health Policy Manag. 2018;7(1):1–9. 29325397 10.15171/ijhpm.2017.90 PMC5745862

[23] Love E. Confronting Islamophobia in the United States: framing civil rights activism among Middle Eastern Americans. In: *Anti-Muslim P rejudice.* Routledge; 2013:191–215. 10.4324/9781315875866.

[24] Ludvigsson JF, Otterblad-Olausson P, Pettersson BU, Ekbom A. The Swedish personal identity number: possibilities and pitfalls in healthcare and medical research. Eur J Epidemiol. 2009;24(11):659–67. 19504049 10.1007/s10654-009-9350-y PMC2773709

[25] Ludvigsson JF, Andersson E, Ekbom A, et al. External review and validation of the Swedish National inpatient register. BMC Public Health. 2011;11:450. 21658213 10.1186/1471-2458-11-450 PMC3142234

[26] Kuitunen I, Ponkilainen VT, Uimonen MM, Eskelinen A, Reito A. Testing the proportional hazards assumption in Cox regression and dealing with possible non-proportionality in total joint arthroplasty research: methodological perspectives and review. BMC Musculoskelet Disord. 2021;22(1):489. 34049528 10.1186/s12891-021-04379-2 PMC8161573

[27] Hess KR. Graphical methods for assessing violations of the proportional hazards assumption in Cox regression. Stat Med. 1995;14(15):1707–23. 7481205 10.1002/sim.4780141510

[28] Braveman P. The social determinants of health and health disparities. Oxford University Press; 2023.

[29] Lantz PM, House JS, Lepkowski JM, Williams DR, Mero RP, Chen J. Socioeconomic factors, health behaviors, and mortality: results from a nationally representative prospective study of US adults. JAMA. 1998;279(21):1703–8. 9624022 10.1001/jama.279.21.1703

[30] House JS, Lantz PM, Herd P. Continuity and change in the social stratification of aging and health over the life course: evidence from a nationally representative longitudinal study from 1986 to 2001/2002 (Americans’ changing lives Study). J Gerontol B Psychol Sci Soc Sci. 2005;60(SpecialIssue2):S15-26. 10.1093/geronb/60.special_issue_2.s15 16251586

[31] Ross CE, Wu C-L. Education, age, and the cumulative advantage in health. J Health Soc Behav. 1996. 10.2307/2137234. 8820314

[32] Mirowsky J, Ross CE. Education, health, and the default American lifestyle. J Health Soc Behav. 2015;56(3):297–306. 26272989 10.1177/0022146515594814

[33] Singh-Manoux A, Richards M, Marmot M. Socioeconomic position across the lifecourse: how does it relate to cognitive function in mid-life? Ann Epidemiol. 2005;15(8):572–8. 16118001 10.1016/j.annepidem.2004.10.007

[34] Masters RK, Link BG, Phelan JC. Trends in education gradients of ‘preventable’mortality: a test of fundamental cause theory. Soc Sci Med. 2015;127:19–28. 25556675 10.1016/j.socscimed.2014.10.023 PMC4420623

[35] Phelan JC, Link BG, Diez-Roux A, Kawachi I, Levin B. Fundamental causes of social inequalities in mortality: a test of the theory. J Health Soc Behav. 2004;45(3):265–85. 15595507 10.1177/002214650404500303

[36] Marmot M. Economic and social determinants of disease. Bull World Health Organ. 2001;79(10):988–9. 11693982 PMC2566682

[37] Marmot M. The health gap: the challenge of an unequal world. Lancet. 2015;386(10011):2442–4. 26364261 10.1016/S0140-6736(15)00150-6

[38] Ferrante F. Education, aspirations and life satisfaction. Kyklos. 2009;62(4):542–62.

[39] Cobb S, Javanbakht A, Khalifeh Soltani E, Bazargan M, Assari S. Racial difference in the relationship between health and happiness in the united States. Psychol Res Behav Manag. 2020;13:481–90. 32547270 10.2147/PRBM.S248633 PMC7259486

[40] Maharlouei N, Cobb S, Bazargan M, Assari S. Subjective health and happiness in the United States: gender differences in the effects of socioeconomic status indicators. J Ment Health Clin Psychol. 2020;4(2):8–17. 32568256 10.29245/2578-2959/2020/2.1196 PMC7304555

[41] Assari S. Income and mental well-being of middle-aged and older Americans: immigrants’ diminished returns. Int J Travel Med Glob Health. 2020;8(1):37–43. 32266301 10.34172/ijtmgh.2020.06

[42] Assari S. Socioeconomic status and current cigarette smoking status: immigrants’ diminished returns. Int J Travel Med Glob Health. 2020;8(2):66–72. 32656271 10.34172/IJTMGH.2020.11

[43] Assari S. Household income and children’s depressive symptoms: immigrants’ diminished returns. Int J Travel Med Glob Health. 2020;8(4):157–64. 34036110 10.34172/IJTMGH.2020.27

[44] Assari S, Akhlaghipour G, Boyce S, Bazargan M, Caldwell CH. Parental Human Capital and Adolescents' Executive Function: Immigrants' Diminished Returns. Med Res Arch. 2020;8(10):10.18103/mra.v8i10.2235. 10.18103/mra.v8i10.2235. 10.18103/mra.v8i10.2235 PMC7695233 33251336

[45] Assari S, Cobb S, Cuevas AG, Bazargan M. Diminished health returns of educational attainment among immigrant adults in the united States. Front Psychiatry. 2020;11:535624. 33329080 10.3389/fpsyt.2020.535624 PMC7728619

[46] Assari S, Perez MU, Johnson N, et al. Education level and self-rated health in the United States: immigrants’ diminished returns. Int J Travel Med Glob Health. 2020;8(3):116–23. 32905455 10.34172/ijtmgh.2020.20

[47] Keyes CL. The black–white paradox in health: flourishing in the face of social inequality and discrimination. J Pers. 2009;77(6):1677–706. 19796064 10.1111/j.1467-6494.2009.00597.x

[48] Markides KS, Rote S. Immigrant Health Paradox. In Emerging Trends in the Social and Behavioral Sciences (eds R.A. Scott and S.M. Kosslyn). 2015. 10.1002/9781118900772.etrds0174.

[49] Williams DR, Mohammed SA. Discrimination and racial disparities in health: evidence and needed research. J Behav Med. 2009;32(1):20–47. 19030981 10.1007/s10865-008-9185-0 PMC2821669

[50] Gómez JM, Gobin RL, Barnes ML, Discrimination. Violence, & healing within marginalized communities. J Trauma Dissociation. 2021;22(2):135–40. 33656410 10.1080/15299732.2021.1869059 PMC8582002

[51] Assari S. Blacks’ diminished return of education attainment on subjective health; mediating effect of income. Brain Sci. 2018. 10.3390/brainsci8090176. 10.3390/brainsci8090176 PMC6162786 30213135

[52] Assari S. The benefits of higher income in protecting against chronic medical conditions are smaller for African Americans than Whites. Healthcare. 2018. 10.3390/healthcare6010002. 10.3390/healthcare6010002 PMC5872209 29315227

[53] Assari S, Lankarani MM. Race and urbanity alter the protective effect of education but not income on mortality. Front Public Health. 2016;4:100. 27242992 10.3389/fpubh.2016.00100 PMC4873510

[54] Assari S, Caldwell CH, Zimmerman MA. Family structure and subsequent anxiety symptoms; minorities’ diminished return. Brain Sci. 2018. 10.3390/brainsci8060097. 10.3390/brainsci8060097 PMC6025006 29857488

[55] Assari S. Ethnicity, educational attainment, and physical health of older adults in the United States. Aging Med. 2019;2(2):104–11. 10.1002/agm2.12050 PMC6788632 31608316

[56] Assari S, Lapeyrouse LM, Neighbors HW. Income and self-rated mental health: diminished returns for high income Black Americans. Behav Sci. 2018;8(5):50. 10.3390/bs8050050. 10.3390/bs8050050 PMC5981244 29772799

[57] Assari S. Family income reduces risk of obesity for white but not black children. Children (Basel). 2018. 10.3390/children5060073. 29890778 10.3390/children5060073 PMC6025246

[58] Assari S, Moghani Lankarani M. Poverty status and childhood asthma in White and Black families: national survey of children’s health. Healthcare. 2018;6(2): 62. 10.3390/healthcare6020062. 10.3390/healthcare6020062 PMC6023379 29895767

[59] Assari S, Caldwell CH. Family income at birth and risk of attention deficit hyperactivity disorder at age 15: racial differences. Children (Basel). 2019;6(1):10. 10.3390/children6010010. 10.3390/children6010010 PMC6352113 30646634

[60] Assari S, Bazargan M. Minorities’ diminished returns of educational attainment on hospitalization risk: National Health Interview Survey (NHIS). Hosp Pract Res. 2019. 10.15171/hpr.2019.17. 31650101 10.15171/HPR.2019.17 PMC6812545

[61] Assari S, Mistry R, Bazargan M, Race. Educational attainment, and E-Cigarette use. J Med Res Innov. 2020;4(1):e000185 10.32892/jmri.185 PMC7034862 32090188

[62] Assari S, Bazargan M. Education level and cigarette smoking: diminished returns of lesbian, gay and bisexual individuals. Behav Sci. 2019;9(10):103. 10.3390/bs9100103. 10.3390/bs9100103 PMC6826997 31554198

[63] Assari S, Bazargan M. Protective effects of educational attainment against cigarette smoking; diminished returns of American Indians and Alaska natives in the National health interview survey. Int J Travel Med Glob Health. 2019. 10.15171/ijtmgh.2019.22. 10.15171/IJTMGH.2019.22 PMC6879009 31772950

[64] Assari S, Bazargan M. Unequal effects of educational attainment on workplace exposure to second-hand smoke by race and ethnicity; minorities’ diminished returns in the National Health Interview Survey (NHIS). J Med Res Innov. 2019. 10.32892/jmri.179. 10.32892/jmri.179 PMC6688774 31404444

[65] Assari S, Mistry R. Diminished return of employment on ever smoking among Hispanic whites in Los Angeles. Health Equity. 2019;3(1):138–44. 31289772 10.1089/heq.2018.0070 PMC6608689

[66] Assari SBM. Second-hand exposure home Second-Hand smoke exposure at home in the united states; minorities’ diminished returns. Int J Travel Med Glob Health 2019;7(3). 10.15171/IJTMGH.2019.28 32195278

[67] Assari S, Farokhnia M, Mistry R. Education attainment and alcohol binge drinking: diminished returns of Hispanics in Los Angeles. Behav Sci. 2019. 10.3390/bs9010009. 30646592 10.3390/bs9010009 PMC6359422

[68] Assari S, Lankarani MM. Education and alcohol consumption among older Americans; Black-White differences. Front Public Health. 2016;4:67. 27148514 10.3389/fpubh.2016.00067 PMC4838609

[69] Assari S, Lankarani MM. Educational attainment promotes fruit and vegetable intake for Whites but not Blacks. J (Basel). 2018;1(1):29–41. 31844842 10.3390/j1010005 PMC6914217

[70] Assari S. Educational attainment and exercise frequency in American women; blacks’ diminished returns. Womens Health Bull. 2019;6(3): e87413. 31552286 10.5812/whb.87413 PMC6757331

[71] Assari S, Najand B. Anti-immigrant sentiments and immigrants’ happiness. Int J Travel Med Glob Health. 2023;11(3):369–81.

[72] Assari S, Boyce S, Bazargan M, Caldwell CH. Diminished returns of parental education in terms of youth school performance: ruling out regression toward the mean. Children (Basel). 2020;7(7): 74. 32645933 10.3390/children7070074 PMC7401872

[73] Osooli M, Ohlsson H, Sundquist J, Sundquist K. Attention deficit hyperactivity disorder in first-and second-generation immigrant children and adolescents: a nationwide cohort study in Sweden. J Psychosom Res. 2021;141: 110330. 33326861 10.1016/j.jpsychores.2020.110330

[74] Osooli M, Ohlsson H, Sundquist J, Sundquist K. Conduct disorder in immigrant children and adolescents: a nationwide cohort study in Sweden. Int J Environ Res Public Health. 2021;18(20): 10643. 34682389 10.3390/ijerph182010643 PMC8535976

[75] Fawcett J. An overview of mood disorders in the DSM-5. Curr Psychiatry Rep. 2010;12:531–8. 20927611 10.1007/s11920-010-0154-2

