# Supplementary material for: Immigrants’ diminished protective effects of parental education and employment on youth mood disorders in Sweden
Source: BMC Psychiatry. 2025 Aug 20;25:800. doi: 10.1186/s12888-025-07264-7 (PMC12366212; doi:10.1186/s12888-025-07264-7)
Supplement: Supplementary file 1 — Supplementary Material 1. [file 12888_2025_7264_MOESM1_ESM.docx]

# Appendixes

| **Appendix Table 1.** The impact of highest parental education on mood disorder in the offspring of natives and immigrants in Sweden. The results are based on ordinary least squares (OLS) regression. | | |  |
| --- | --- | --- | --- |
|  | **Model 1A, β (95% CI)** | **Model 1B, β (95% CI)** | |
| **Sex** |  |  | |
| Females | Reference | Reference | |
| Males | **-3.54% (-3.59; -3.49)** | **-3.54% (-3.59; -3.49)** | |
| **Year of Birth** | **0.38% (0.37; 0.38)** | **0.38% (0.37; 0.38)** | |
| **Immigration background** |  |  | |
| Natives | Reference | Reference | |
| Swedish born with 1 foreign born parent | **0.99% (0.90; 1.08)** | **1.01% (0.84; 1.17)** | |
| Swedish born with foreign born parents | **-2.23% (-2-33; 2-13)** | **-2.71% (-2.86; -2.56)** | |
| Born outside Sweden | **-3.01% (-3.11; -2.91)** | **-3.88% (-4.05; -3.71)** | |
| **Highest parental education** |  |  | |
| Low Education | Reference | Reference | |
| Mid Education | **-0.28% (-0.35; -0.20)** | -**0.41% (-0.50; -0.32)** | |
| High Education | **-0.99% (-1.05;-0.93)** | -1.21% (-1.28; 1.14) | |
| **Interaction terms** |  |  | |
| Swedish born with 1 foreign born parent* Mid Education | - | -0.09% (-0.35; 0.18) | |
| Swedish born with 1 foreign born parent* High Education | - | 0.00% (-0.21; 0.21) | |
| Swedish born with foreign born parents* Mid Education | - | 0.06% (-0.20; 0.32) | |
| Swedish born with foreign born parents* High Education | - | **1.16% (0.94; 1.38)** | |
| Born outside Sweden * Mid Education | - | **1.22% (0.95; 1.48)** | |
| Born outside Sweden * High Education | - | **1.42% (1.19; 1.65)** | |
|  |  |  | |
|  |  |  | |

| **Appendix Table 2.** The impact of residing with both parents on mood disorder in the offspring of natives and immigrants in Sweden. The results are based on ordinary least squares (OLS) regression. | | |
| --- | --- | --- |
|  | **Model 2A, β** **(95% CI)** | **Model 2B, β (95% CI)** |
| **Sex** |  |  |
| Females | Reference | Reference |
| Males | **-3.54% (-3.59; -3.48)** | **-3.54% (-3.59; -3.48)** |
| **Year of Birth** | **0.37% (0.37; 0.38)** | **0.37% (0.37; 0.38)** |
| **Immigration background** |  |  |
| Natives | Reference | Reference |
| Swedish born with 1 foreign born parent | **0.63% (0.54; 0.72)** | **0.81% (0.67; 0.96)** |
| Swedish born with foreign born parents | **-2.15% (-2.25; -2.05)** | **-2.78% (-2.95; -2.60)** |
| Born outside Sweden | **-2.40% (-2.50; -2.30)** | **-3.76% (-4.03; -3.49)** |
| **Residing with parents** |  |  |
| Residing with one parent | Reference | Reference |
| Residing with Both parents | **-3.17% (-3.23; -3.11)** | **-3.28% (-3.35; -3.21)** |
| **Interaction terms** |  |  |
| Swedish born with 1 foreign born parent * Residing with Both parents | - | **-0.33% (-0.52; -0.15)** |
| Swedish born with foreign born parents * Residing with Both parents | - | **0.90% (0.69; 1.11)** |
| Born outside Sweden * Residing with Both parents | - | **1.59% (1.30; 1.88)** |
|  |  |  |

| **Appendix Table 3.** Logistic regression assessing the impact of parental occupation on mood disorder in the offspring of natives and immigrants in Sweden. The results are based on ordinary least squares (OLS) regression. | | |
| --- | --- | --- |
|  | **Model 3A, β (95% CI)** | **Model 3B, β (95% CI)** |
| **Sex** |  |  |
| Females | Reference | Reference |
| Males vs | **-3.54% (-3.59; -3.49)** | **-3.54% (-3.59; -3.49)** |
| **Year of Birth** | **0.37% (0.37; 0.38)** | **0.37% (0.37; 0.38)** |
| **Immigration background** |  |  |
| Natives | Reference | Reference |
| Swedish born with 1 foreign born parent | **0.84% (0.75; 0.93)** | **1.39% (1.24; 1.54)** |
| Swedish born with foreign born parents | -2.42% (-2.52; 2.32) | -**1.89% (-2.03; -1.74)** |
| Born outside Sweden | **-3.38% (-3.48; -3.28)** | **-3.17% (-3.30; -3.04)** |
| **Parental work status** |  |  |
| None parent working | Reference | Reference |
| One parent working | **0.82% (0.74; 0.90)** | **1.48% (1.37; 1.59)** |
| Both parents working | **-0.99% (-1.05; -0.93)** | **-0.88% (-0.94; -0.81)** |
| **Interactions** |  |  |
| Swedish born with 1 foreign born parent* One parent working | - | **-1.29% (-1.56; -1.02)** |
| Swedish born with 1 foreign born parent* Both parents working | - | **-0.77% (-0.97; -0.57)** |
| Swedish born with foreign born parents* One parent working | - | **-1.98% (-2.24; -1.72)** |
| Swedish born with foreign born parents* Both parents working | - | **-0.46% (-0.68; -0.24)** |
| Born outside Sweden * One parent working | - | **-2.03% (-2.29; -1.77)** |
| Born outside Sweden * Both parents working | - | **1.26% (0.98; 1.54)** |
|  |  |  |

| **Appendix Table 4.** The impact of income on mood disorder in the offspring of natives and immigrants in Sweden. The results are based on ordinary least squares (OLS) regression. | | |
| --- | --- | --- |
|  | **Model 4A, β (95% CI)** | **Model 4B, β (95% CI)** |
| **Sex** |  |  |
| Females | Reference | Reference |
| Males | **-3.55% (-3.60; -3.49)** | **-3.55% (-3.60; -3.49)** |
| **Year of Birth** | **0.44% (0.44; 0.45)** | **0.44% (0.44; 0.45)** |
| **Immigration background** |  |  |
| Natives | Reference | Reference |
| Swedish born with 1 foreign born parent | **0.70% (0.61; 0.79)** | **0.60% (0.42; 0.77)** |
| Swedish born with foreign born parents | **-2.69% (-2.79; -2.59)** | **-3.00% (-3.17; -2.82)** |
| Born outside Sweden | **-4.12% (-4.22; -4.01)** | **-4.80% (-4.94; -4.65)** |
| **Family income** |  |  |
| Low Income | Reference | Reference |
| Mid-Low Income | **-1.37% (-1.44; -1.29)** | **-1.63% (-1.72; -1.53)** |
| Mid-High Income | **-2.09% (-2.17; -2.02)** | **-2.24% (-2.33; -2.15)** |
| High Income | **-3.60% (-3.68, -3.52)** | **-3.78% (-3.88; -3.69)** |
| **Interactions** |  |  |
| Swedish born with 1 foreign born parent* Mid-Low Income | - | **0.30% (0.04; 0.55)** |
| Swedish born with 1 foreign born parent* Mid-High Income | - | 0.02% (-0.23; 0.28) |
| Swedish born with 1 foreign born parent* High Income | - | 0.05% (-0.20; 0.30) |
| Swedish born with foreign born parents* Mid-Low Income | - | **0.59% (0.33; 0.84)** |
| Swedish born with foreign born parents* Mid-High Income | - | 0.20% (-0.07; 0.47) |
| Swedish born with foreign born parents* High Income | - | **0.44% (0.14; 0.74)** |
| Born outside Sweden * Mid-Low Income | - | **1.36% (1.12; 1.60)** |
| Born outside Sweden * Mid-High Income | - | **0.91% (0.59; 1.23)** |
| Born outside Sweden * High Income | - | **2.16% (1.73; 2.59)** |
|  |  |  |

| **Appendix Table 5.** The impact of family socioeconomic status (SES) and family composition different immigrant groups on mood disorder in the offspring of natives and immigrants in Sweden with all indicators included in the same model. The results are based on ordinary least squares (OLS) regression. | | |
| --- | --- | --- |
|  | **Model 5A, β (95% CI)** | **Model 5B, β (95% CI)** |
| **Sex** |  |  |
| Females | Reference | Reference |
| Males | **-3.54% (-3.59; -3.49)** | **-3.54% (-3.59; -3.49)** |
| **Year of Birth** | **0.41% (0.41; 0.42)** | **0.41% (0.41; 0.42)** |
| **Immigration background** |  |  |
| Natives | Reference | Reference |
| Swedish born with 1 foreign born parent | **0.51% (0.42; 0.60)** | **1.03% (0.79; 1.27)** |
| Swedish born with foreign born parents | **-2.60% (-2.70; -2.50)** | -2.75% (-2.99; -0) |
| Born outside Sweden | **-3.24% (-3.35; -3.13)** | **-4.17% (-4.50; -3.84)** |
| **Highest parental education** |  |  |
| Low Education | Reference | Reference |
| Mid Education | -0.04% (-0.12; 0.03) | -0.14% (-0.23; -0.05) |
| High Education | **-0.25% (-0.32; -0.19)** | **-0.40% (-0.47; -0.32)** |
| **Residing with parents** |  |  |
| Residing with one parent | Reference | Reference |
| Residing with Both parents | **-2.60% (-2.67; -2.53)** | **-2.67% (-2.75; -2.58)** |
| **Parental work status** |  |  |
| None parent working | Reference | Reference |
| One parent working | 0.34% (0.26; 0.43) | 0.88% (0.77; 0.99) |
| Both parents working | -0.55% (-0.61; -0.49) | -0.48% (-0.55; -0.41) |
| **Income** |  |  |
| Low Income | Reference | Reference |
| Mid-Low Income | -0.17% (-0.25; -0.09) | -0.14% (-0.24; -0.03) |
| Mid-High Income | -0.40% (-0.49; -0.31) | -0.32% (-0.34; -0.12) |
| High Income | -1.44% (-1.54; -1.34) | -1.30% (-1.42; -1.19) |
| **Interactions** |  |  |
| Swedish born with 1 foreign born parent* Mid Education | - | -0.01% (-0.28; 0.26) |
| Swedish born with 1 foreign born parent* High Education | - | 0.08% (-0.14; 0.29) |
| Swedish born with foreign born parents* Mid Education | - | -0.01% (-0.27; 0.26) |
| Swedish born with foreign born parents* High Education | - | 0.78% (0.56; 1.00) |
| Born outside Sweden * Mid Education | - | 0.82% (0.55; 1.09) |
| Born outside Sweden * High Education | - | 0.74% (0.50; 0.97) |
|  |  |  |
| Swedish born with 1 foreign born parent * Residing with Both parents | - | **-0.49% (-0.17; -0.26)** |
| Swedish born with foreign born parents * Residing with Both parents | - | **0.88% (0.65; 1.12)** |
| Born outside Sweden * Residing with Both parents | - | **0.79% (0.48; 1.11)** |
|  |  |  |
| Swedish born with 1 foreign born parent* One parent working | - | **-1.21% (-1.48; -0.4)** |
| Swedish born with 1 foreign born parent* Both parents working | - | **-0.52% (-0.72; -0.31)** |
| Swedish born with foreign born parents* One parent working | - | **-1.54% (-1.81; -1.28)** |
| Swedish born with foreign born parents* Both parents working | - | -0.23% (-0.46; 0.01) |
| Born outside Sweden * One parent working | - | **-1.36% (-1.63; -1.09)** |
| Born outside Sweden * Both parents working | - | **1.46% (1.15; 1.76)** |
|  |  |  |
| Swedish born with 1 foreign born parent* Mid-Low Income | - | 0.26% (-0.01; 0.53) |
| Swedish born with 1 foreign born parent* Mid-High Income | - | 0.14% (-0.16; 0.43) |
| Swedish born with 1 foreign born parent* High Income | - | **0.32% (0.01; 0.62)** |
| Swedish born with foreign born parents* Mid-Low Income | - | -0.26% (-0.53; 0.01) |
| Swedish born with foreign born parents* Mid-High Income | - | **-0.83% (-1.13; -0.54)** |
| Swedish born with foreign born parents* High Income | - | **-0.83% (-1.18; -0.49)** |
| Born outside Sweden * Mid-Low Income | - | -0.09% (-0.34; 0.16) |
| Born outside Sweden * Mid-High Income | - | **-1.04% (-1.39; -0.70)** |
| Born outside Sweden * High Income | - | -0.37% (-0.82; 0.08) |
|  |  |  |
